# Activated T follicular helper-like cells are released into blood after oral vaccination and correlate with vaccine specific mucosal B-cell memory

**DOI:** 10.1038/s41598-018-20740-3

**Published:** 2018-02-09

**Authors:** Ana Cárdeno, Maria K. Magnusson, Marianne Quiding-Järbrink, Anna Lundgren

**Affiliations:** Department of Microbiology and Immunology, University of Gothenburg, Gothenburg, Sweden

## Abstract

T follicular helper (Tfh)-like cells with potent B-cell helping ability are mobilized into human circulation after parenteral vaccination and are generally held to reflect ongoing germinal center reactions. However, whether mucosal vaccination induces systemic Tfh responses and how such responses may relate to IgA production are unknown. We investigated the frequencies, phenotype and function of circulating Tfh-like CD4^+^CXCR5^+^ T cells (cTfh) in adults receiving an oral inactivated enterotoxigenic *Escherichia coli* vaccine. Subjects were classified as vaccine responders or weak/non-responders based on their intestine-derived antibody-secreting cell (ASC) IgA responses to major vaccine antigens. Oral immunization induced significantly increased proportions of cTfh cells expressing the cTfh activation marker inducible costimulator (ICOS) in ASC responders, but not in weak/non-responders. Vaccination also enhanced the expression of IL-21, Th17 markers and integrin β7 by activated cTfh cells, supporting functionality and gut homing potential. cTfh cells promoted total and vaccine specific IgA production from cocultured B cells. Magnitudes of cTfh responses assessed within a week after primary vaccinations correlated with memory intestine-derived vaccine specific IgA responses 1–2 years later. We conclude that activated ICOS^+^ Tfh-like cells are mobilized into blood after oral vaccination and may be used as biomarkers of vaccine specific mucosal memory in humans.

## Introduction

Protection against non-invasive enteric infections such as cholera and enterotoxigenic *Escherichia coli* (ETEC) diarrhoea is mainly mediated through antigen-specific secretory IgA (SIgA) antibodies produced locally in the mucosa^1,2^. Such antibodies can be induced both by natural infection and by oral vaccination^1,2^. However, little is currently known about the longevity of mucosal IgA responses in humans, including how such responses are initiated and maintained on a cellular level. This is largely due to the limited access to mucosal samples and scarcity of validated methods for measuring immunological memory in humans. Therefore, identification of easily assessable biomarkers associated with induction of protective and long-lasting IgA responses may improve our understanding of mucosal immunity in humans and facilitate the development and evaluation of new mucosal vaccines.

CD4^+^ T follicular helper (Tfh) cells promote long-lived humoral immunity by providing help to B cells in germinal centers (GCs) in secondary lymphoid organs^3–5^. Although more fully characterized in mice, Tfh cells with potent B cell helping ability have also been observed in human tonsils^6–8^. GC Tfh cells express the chemokine receptor CXCR5, which guides their migration into B cell follicles in response to the CXCL13 ligand, as well as inducible costimulator (ICOS), a molecule essential for the secretion of IL-21, which potently promotes class-switching and B cell differentiation into plasma cells and memory B cells^9–11^. So far, the evaluation of Tfh responses in humans has been hampered by the difficulty to obtain secondary lymphoid tissue. However, a subset of circulating CD4^+^CXCR5^+^ T cells which shares both phenotypic and functional properties with GC Tfh cells has been identified in both humans and mice^10,12,13^. Although the vast majority of these circulating Tfh-like (cTfh) cells are in a resting state, studies show that ongoing GC reactions in peripheral lymph nodes result in the emergence of activated CD4^+^CXCR5^+^ cTfh cells, characterized by high expression of ICOS and programmed cell death protein 1 (PD-1) and low expression of CCR7, in peripheral blood^10,13^. Furthermore, the cTfh responses appear to reflect the development of protective high affinity antibody responses after parenteral immunization in both mice and humans^13–15^. However, little is currently known about Tfh responses in GC or blood during mucosal immune responses in humans and whether cTfh responses can be used to monitor the processes leading to generation of long-lasting and protective mucosal IgA responses.

We have recently demonstrated the capacity of a novel oral inactivated whole cell vaccine against ETEC diarrhoea (ETVAX) to induce both primary and memory mucosal IgA responses in two large Phase I trials in Swedish adults, using vaccine-specific IgA antibody secreting cell (ASC) responses in peripheral blood as well as SIgA antibody levels in fecal extracts as correlates of intestinal SIgA responses^16,17^. In these trials, we showed that the multivalent ETEC vaccine, consisting of four inactivated recombinant *E*. *coli* strains over-expressing the major ETEC colonization factors (CFs) CFA/I, CS3, CS5 and CS6 mixed with a heat labile toxin binding-subunit (LTB) related toxoid, LCTB*A*^18^, was safe and induced mucosal IgA responses to all CFs and to LTB in a majority of subjects^16^. Importantly, we also demonstrated that the vaccine induced long-lasting IgA responses, since a late booster dose administered 1–2 years after primary vaccinations induced significantly more rapid blood ASC responses than a single dose vaccination given to naïve subjects^17^. Administration of the vaccine with the mucosal adjuvant double mutant heat labile toxin (dmLT) enhanced primary IgA ASC responses to antigens present in low amounts in the vaccine, but did not influence memory B cell responses^16,17^. In this study we utilized blood samples collected in the two previously reported vaccine trials for exploratory analyses to determine if oral vaccination gives rise to cTfh responses. We also investigated the potential relation between cTfh responses and memory IgA responses elicited in the intestinal mucosa.

## Results

### Oral vaccination induced cTfh responses

To determine if oral vaccination induces release of activated Tfh-like cells into peripheral blood, we selected frozen peripheral blood mononuclear cell (PBMC) samples from a subset of ETEC vaccinees, including subjects who had responded strongly as well as weakly/not at all to the five major vaccine antigens (CFA/I, CS3, CS5, CS6 and LTB) in the antibodies in lymphocyte supernatants (ALS) assay used for ASC assessment after primary and booster vaccinations (See Supplementary Fig. S1 and Materials and Methods for detailed study design and responder criteria)^16,17^. Blood ASCs induced by oral vaccination express the gut homing marker integrin α4β7 and correlate with ASC responses in intestinal mucosa as well as with IgA responses in fecal and lavage samples and are thus good surrogate measures of mucosal immune responses^19–24^. In the ALS assay, *ex vivo* secreted antibodies from plasma blasts/plasma cells (i.e. ASCs) are analyzed in culture supernatants, and provide comparable results to the more traditional ELISPOT assay for assessment of intestine-derived ASC responses to oral vaccination^16,25–28^. Subjects were defined as weak/non-responders to the vaccine based on the combined ALS response index, defined as the sum of the magnitudes (maximal fold rises above prevaccination baseline IgA levels) of IgA ALS responses to the five major vaccine antigens (Supplementary Fig. S1).

**Figure 1:**
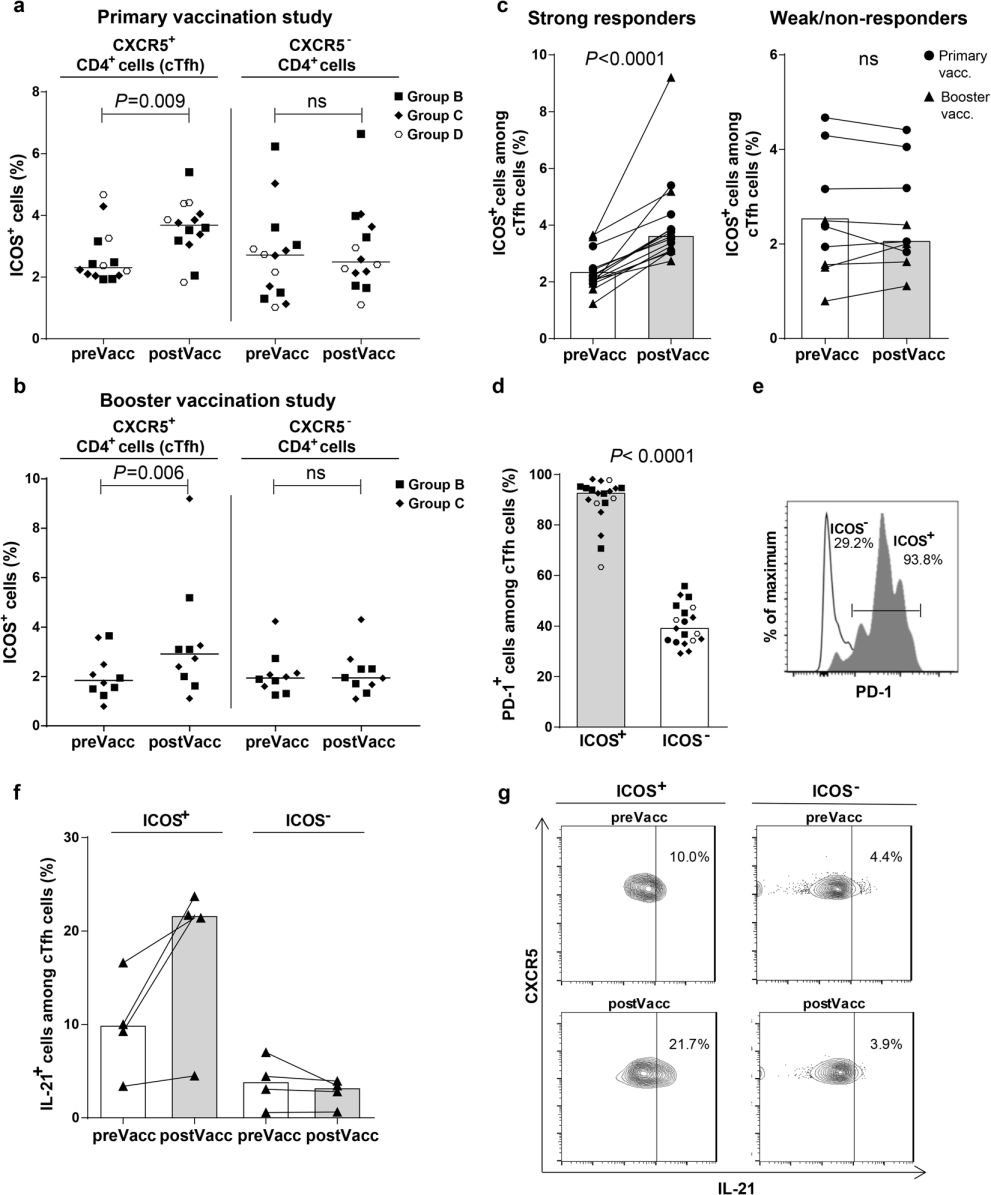
Oral ETEC vaccination induced increased frequencies of activated Tfh-like cells in peripheral blood. (**a**–**c**) ICOS expression was analysed in PBMCs by FCM before (preVacc) and after (postVacc) primary and booster ETEC vaccinations. Strong ALS responders and weak/non-responders were randomly selected from each vaccination group (Group B; vaccine alone and groups C and D; vaccine with 10 or 25 µg dmLT, respectively, in the primary vaccination study; all booster vaccinations were given without adjuvant). (**a** and **b**) Frequencies of ICOS^+^ cells among CD4^+^CXCR5^+^ cTfh and CD4^+^CXCR5^−^ cells in the primary (**a**, n = 14) and booster (**b**, n = 10) vaccination studies (separate sets of individuals). (**c**) Frequencies of ICOS^+^ cells among cTfh in strong versus weak/non-responders (data pooled from the primary and booster vaccination study, n = 24). (**d**) PD-1 expression in ICOS^+^ and ICOS^−^ cTfh cells in post vaccination samples (symbols as in Fig. 1a and **b**, n = 19). (**e**) Representative PD-1 staining among ICOS^+^ and ICOS^−^ cTfh cells. (**f**) Frequencies of IL-21-producing cells among ICOS^+^ and ICOS^−^ cTfh cells isolated from 4 strong ALS responders after stimulation of PBMCs with PMA and ionomycin. (**g**) Representative staining of IL-21 expression in cTfh cells. (**a–d**, **f**) Each symbol represents one individual. Horizontal lines or bars indicate median values.

Since ICOS has previously been shown to consistently reflect cTfh activation after parenteral vaccination and has been used to monitor cTfh responses in several different studies^14,15,29,30^ we focused our analysis on ICOS-expressing cTfh cells, defined as CD4^+^CXCR5^+^ T cells, in selected samples before and after oral ETEC vaccination by flow cytometric (FCM) analysis (the gating strategy is shown in Supplementary Fig. S2). We found significantly increased proportions of ICOS^+^ cells among CD4^+^CXCR5^+^ cTfh cells in post- compared to prevaccination samples in subjects receiving two primary oral doses of ETEC vaccine, two weeks apart (Fig. 1a). Comparable increases in ICOS expression on cTfh cells were also detected after administration of a single, late booster vaccine dose 1–2 years later (Fig. 1b; data shown is from a separate set of subjects than the primary vaccinations subjects in Fig. 1a). In contrast, the frequencies of ICOS^+^ cells among CD4^+^CXCR5^−^ T cells did not change after vaccination (Fig. 1a and b). Increased ICOS expression on cTfh cells was often detected both after the first (day 7) and second dose (day 5), with a majority (70%) of strong ALS responders to the vaccine having higher responses at the later time point. The ICOS expression decreased in most subjects on day 7 compared to day 5 after the second dose. After administration of the late booster dose, increased ICOS expression compared to pre booster levels was commonly observed both on day 4/5 and day 7, with a majority (60%) of strong ALS responders having higher responses on day 4/5 compared to day 7. Maximal increases in ICOS expression on cTfh cells detected at any time point after primary (one or two doses) or booster vaccinations are indicated in the graphs (Fig. 1a–c). The maximal cTfh responses observed after administration of the late booster dose were of similar magnitudes as the maximal responses after the primary vaccine doses (Fig. 1a and b). We also found a small, but significant increase in CXCR5 expression among CD4^+^ T cells (13.8 vs 15.3%, *P* = 0.02, n = 14) in post- compared to prevaccination samples in subjects receiving two primary oral doses of ETEC vaccine. However, the frequencies of CXCR5^+^ cells did not change in response to booster vaccination 1–2 years later (9.0 vs 9.2%, *P* = 0.2, n = 10).

Since ICOS expression increased to comparable extent both with regard to magnitude of responses and frequencies of responders after primary and booster vaccination, data from the two sets of subjects were pooled and analyzed in relation to vaccine-specific intestine-derived IgA ALS responses (Fig. 1c). cTfh responses, henceforth defined as increased ICOS expression after vaccination compared to prevaccination baseline levels, were primarily detected in subjects classified as strong ALS responders, whereas responses in weak/non-responders were not significant. cTfh responses were observed in subjects receiving primary vaccine doses with as well as without dmLT adjuvant (Group B versus C or D; Fig. 1a). A large majority of the ICOS^+^ cTfh cells expressed the Tfh-associated marker PD-1, consistent with a cTfh phenotype^10,31^, and the PD-1 expression among ICOS^−^ cTfh cells was significantly lower (Fig. 1d and e). The vast majority (>90%) of the ICOS^+^ cTfh cells were CD27^+^CD45RO^+^ central memory cells (Supplementary Fig. S2).

Previous studies show that cTfh cells produce IL-21 after activation and that this cytokine is critical for their ability to aid antibody production^10–12,32^. To analyze the functional properties of cTfh cells in ETEC vaccinees, IL-21 production was analyzed after stimulation of PBMCs with phorbol myristate acetate (PMA) and ionomycin. Consistent with their activated Tfh-like phenotype, ICOS^+^CD4^+^CXCR5^+^ T cells produced IL-21 while ICOS^−^ cells produced little IL-21 (Fig. 1f and g). Notably, ETEC vaccination induced increased expression of IL-21 among ICOS^+^ cTfh cells, but not among ICOS^−^ cTfh cells (Fig. 1f and g), in all tested individuals.

Collectively, these results demonstrate that oral ETEC vaccination induces the appearance of activated ICOS^+^CD4^+^CXCR5^+^ cells with Tfh-like characteristics in peripheral blood after both primary and booster mucosal immunizations in individuals with a strong IgA ASC response to the vaccine.

### cTfh responses correlated with vaccine specific IgA responses after oral vaccination

**Figure 2:**
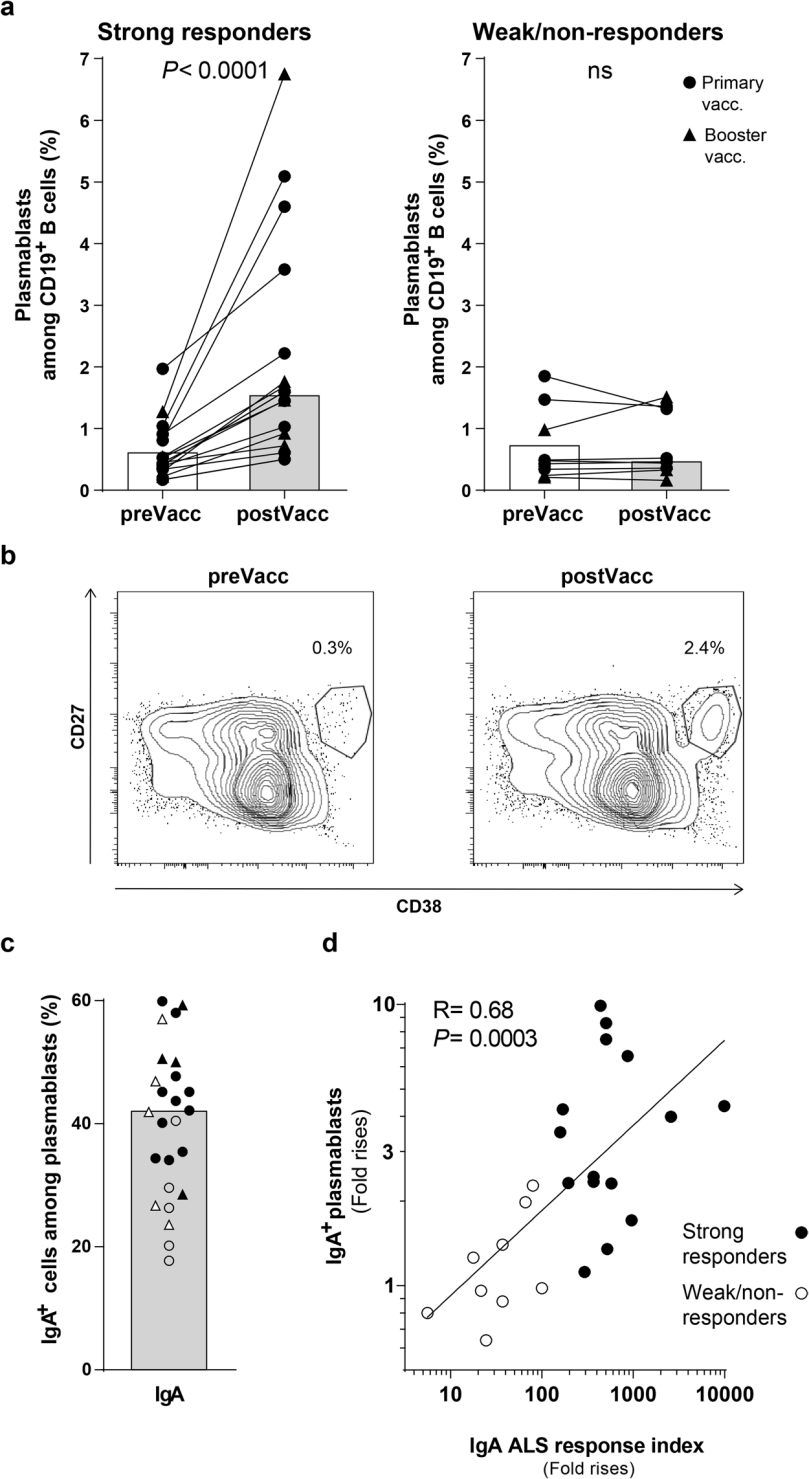
Oral ETEC vaccination induced increased frequencies of circulating plasmablasts. (**a** and **b**) Frequencies of plasmablasts (CD27^+^CD38^hi^) among CD19^+^ B cells in PBMCs collected before (preVacc) and after (postVacc) vaccination were analysed by FCM. (**a**) Data for strong and weak/non-responders (data pooled from the primary and booster vaccination study; the same subjects as in Fig. 1a–c). (**b**) Representative plasmablast staining from one strong ALS responder. (**c**) Frequencies of IgA^+^ cells among plasmablasts in post vaccination samples from strong and weak/non ALS responders from the primary and booster studies (symbols as in (**a**), filled symbols; strong responders and open symbols; weak/non-responders). (**d**) Correlation between the combined response index of vaccine specific IgA ALS responses against the five major vaccine antigens and the magnitudes (fold rises) of IgA^+^ plasmablast responses (n = 24; same subjects as in **a**). (**a**,**c** and **d**) Each symbol represents one individual. (**a** and **c**) Bars indicate median values.

Previous studies show that after parenteral vaccination, activated cTfh cells promote the differentiation of plasmablasts, which emerge in peripheral blood at the same time as activated cTfh cells^14,30,33,34^. Our ALS data indirectly demonstrate that oral ETEC vaccination also induces the release of plasmablasts into peripheral blood, since vaccine specific ASC responses in blood induced by oral vaccination are mainly derived from gut homing plasmablasts^19,23,24^. Consistently, we found significantly increased frequencies of circulating CD27^+^CD38^hi^ plasmablasts in subjects both after primary and booster vaccinations (Supplementary Figs S3 and S4). As would be expected, increased frequencies of plasmablasts were primarily detected in strong ALS responders (Fig. 2a and b, maximal response magnitudes are shown). The kinetics of plasma blast responses, as detected by FCM, was consistent with previous reports of the ALS response kinetics after primary and booster vaccinations^16,17^. Most subjects (75%) had higher responses after the second compared to the first vaccine dose, with maximal responses detected on day 5 after the second dose in a majority of subjects. After the late booster dose, 90% of subjects had higher responses on day 4/5 compared to day 7. The maximal plasmablast responses to the primary vaccine doses and the late booster dose were of similar magnitudes (Figure 2a and Supplementary Fig. S4). In contrast to the observed plasmablast responses, frequencies of CD19^+^ B cells or CD19^+^CD27^+^ memory B cells did not change in response to vaccination (Supplementary Fig. S4). Comparable magnitudes of plasmablast responses were observed after vaccination with or without dmLT (Supplementary Fig. S4). A large proportion of plasmablasts expressed IgA (Fig. 2c). Although the frequencies of total plasmablasts increased after vaccination, the relative proportions of IgA^+^ cells among the plasmablasts did not change. The magnitudes of IgA^+^ plasmablast responses (fold rises in plasmablast frequencies) correlated significantly with the combined magnitudes of vaccine specific IgA ALS responses to the five major vaccine antigens (Fig. 2d). The expression of IgG or IgM on plasmablasts was not analyzed due to limited cell numbers and technical difficulties to analyze such cells.

**Figure 3:**
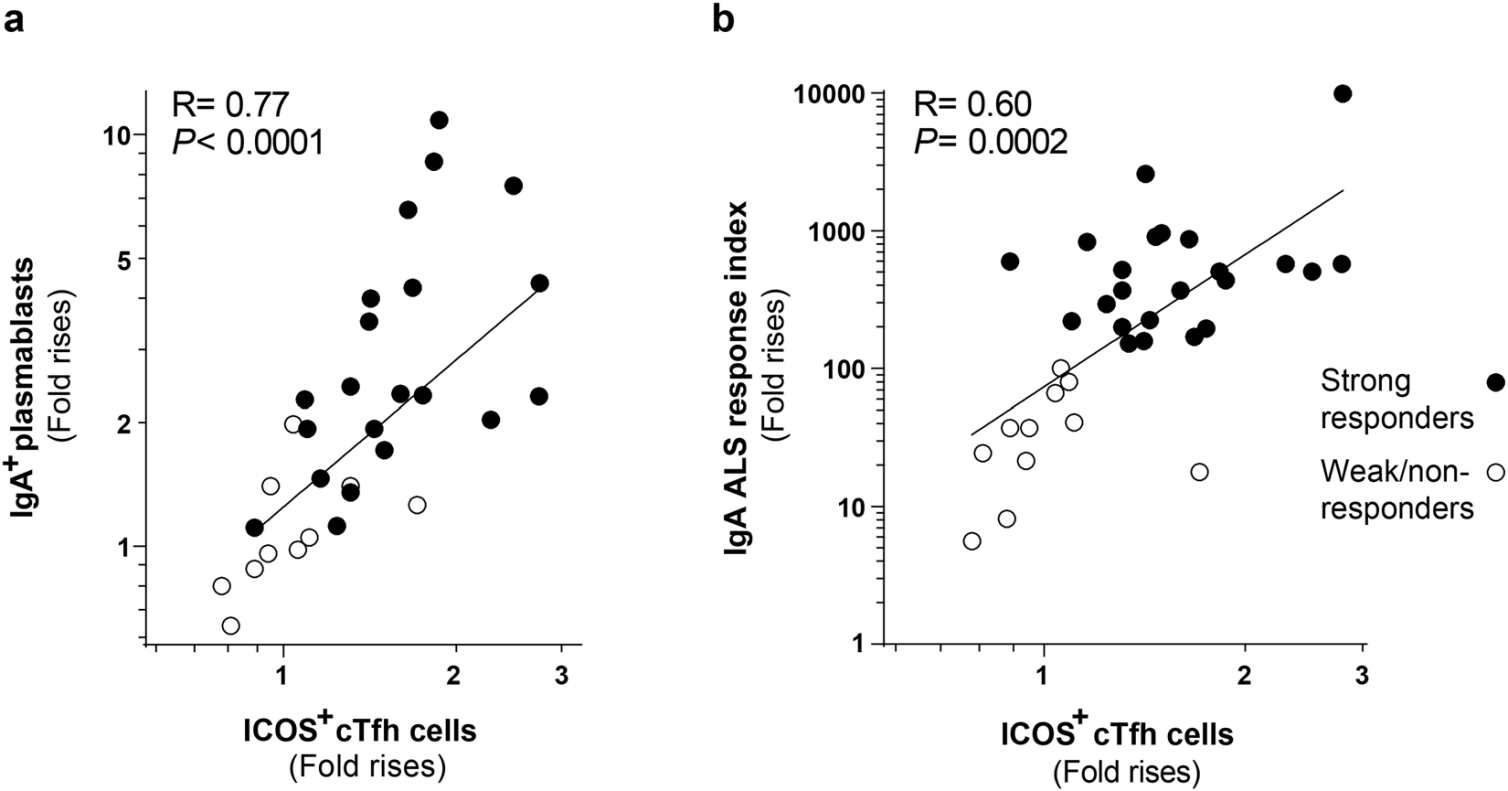
cTfh responses induced by oral ETEC vaccination correlated with blood plasmablast and vaccine specific ALS responses. Correlation between the magnitudes (fold rises) of activated cTfh cell (ICOS^+^CD4^+^CXCR5^+^) and (**a**) IgA^+^ plasmablast (IgA^+^CD27^+^CD38^hi^CD19^+^) responses and (**b**) the combined response index of vaccine specific IgA ALS responses against the five major vaccine antigens. Each symbol represents one individual; n = 31 in (**a**) (18 subjects from the primary and 13 from the booster vaccination study) and n = 34 in (**b**) (20 subjects from the primary and 14 from the booster vaccination study) and data is displayed in a log-log scale plot.

Given the importance of Tfh cells for plasma cell development, we next evaluated the relation between the cTfh and plasmablast and ALS responses induced by ETEC vaccination in a larger number of vaccinees from the primary and booster vaccination studies. The magnitudes of the cTfh responses correlated significantly with both the IgA^+^ plasmablast responses (Fig. 3a, R = 0.77), the total plasma blast responses (R = 0.70, not shown) and the combined index of ALS responses against the five major vaccine antigens (Fig. 3b, R = 0.60).

Taken together, these results demonstrate that IgA^+^ plasmablasts and activated cTfh cells are released into peripheral blood in parallel after oral vaccination, and that the magnitudes of these two types of responses correlate. These data indicate that a functional relationship may exist between cTfh activation and plasmablast induction after oral vaccination.

### cTfh cells from ETEC vaccinees promoted vaccine specific IgA antibody production

**Figure 4:**
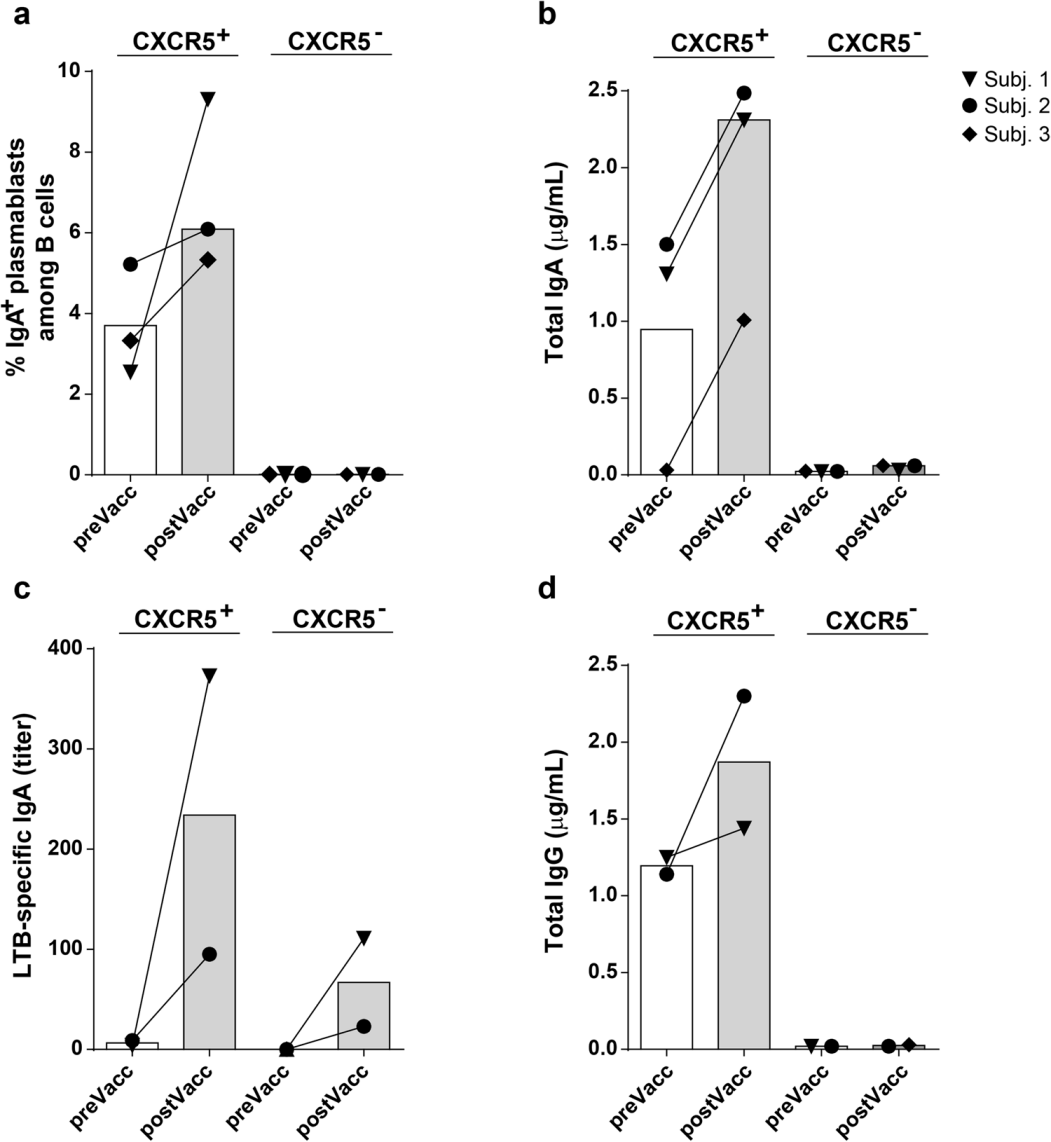
cTfh cells from ETEC vaccinees promoted plasmablast formation and IgA antibody production. CXCR5^+^CD4^+^CD3^+^ cTfh cells, CXCR5^−^CD4^+^CD3^+^ T cells and memory B cells were sorted from PBMCs collected from three strong ALS responders before (preVacc) and after (postVacc) ETEC vaccination. cTfh or CD4^+^CXCR5^−^ cells were coincubated with memory B cells collected at the same time point and stimulated with SEB for 6 days. The frequencies of IgA^+^CD27^+^CD38^hi^ plasmablasts among CD19^+^ B cells (**a**) and the levels of total IgA (**b**), LTB-specific IgA (**c**) and total IgG (**d**) antibodies in culture supernatants were analyzed by FCM (**a**) or ELISA (**b**,**c** **and d**), respectively. Each symbol represents one subject (Subj.) and bars indicate median values (n = 2–3).

To evaluate the functional capacity of cTfh cells from ETEC vaccinees to influence B cell antibody production, interactions between cTfh cells and B cells were mimicked *in vitro* by culturing sorted CD4^+^CXCR5^+^ cTfh or CD4^+^CXCR5^−^ T cells with CD19^+^CD27^+^ memory B cells isolated from the same cell samples from three strong responders to the vaccine (Supplementary Fig. S5). After 6 days of coculture in the presence of the superantigen staphylococcus enterotoxin B (SEB), ≥93% of the T cells were alive. cTfh cells strongly promoted B cell survival and/or expansion, since cultures with CXCR5^+^ cells contained significantly higher proportions of live CD19^+^ B cells than cultures with CXCR5^−^ cells (≥83% *vs* ≤18%). In cultures with cTfh cells memory B cells differentiated into IgA expressing plasmablasts, whereas almost no live IgA^+^ plasmablasts were detected in cultures with CXCR5^−^ cells (Fig. 4a). Increased proportions of live IgA^+^ plasmablasts were present in cultures with cells collected post- compared to prevaccination in all three individuals tested (Fig. 4a). Furthermore, relatively high levels of IgA antibodies were detected in supernatants from cTfh cocultures, and the production increased in response to vaccination in all subjects, whereas much less IgA was detected in cocultures with CXCR5^−^ cells isolated at any time point (Fig. 4b). Due to the low number of cells and small volumes of culture supernatants available from one of the three subjects tested, samples from only two volunteers could be analyzed for vaccine specific IgA antibodies or total IgG antibodies. Importantly, our results from these subjects suggest that cTfh cells could also promote the production of vaccine specific antibodies, since increased levels of LTB-specific IgA antibodies were detected in cocultures with post vaccination memory B cells (Fig. 4c). Some LTB-specific antibodies were also detected in cultures with CXCR5^−^ cells using the highly sensitive GM1-LTB ELISA method, but at about 4-fold lower levels than in cTfh cultures from the same volunteers. IgG antibodies were also primarily detected in cTfh cocultures and levels increased after vaccination (Fig. 4d). Due to the low numbers of weak responders to the vaccine, the functional capacity of cTfh cells from this study group could not be evaluated.

Collectively, these results support the conclusion that cTfh cells from ETEC vaccinees can actively promote plasmablast differentiation and IgA production.

### Activated cTfh cells and plasmablasts induced by oral vaccination expressed integrin β7

**Figure 5:**
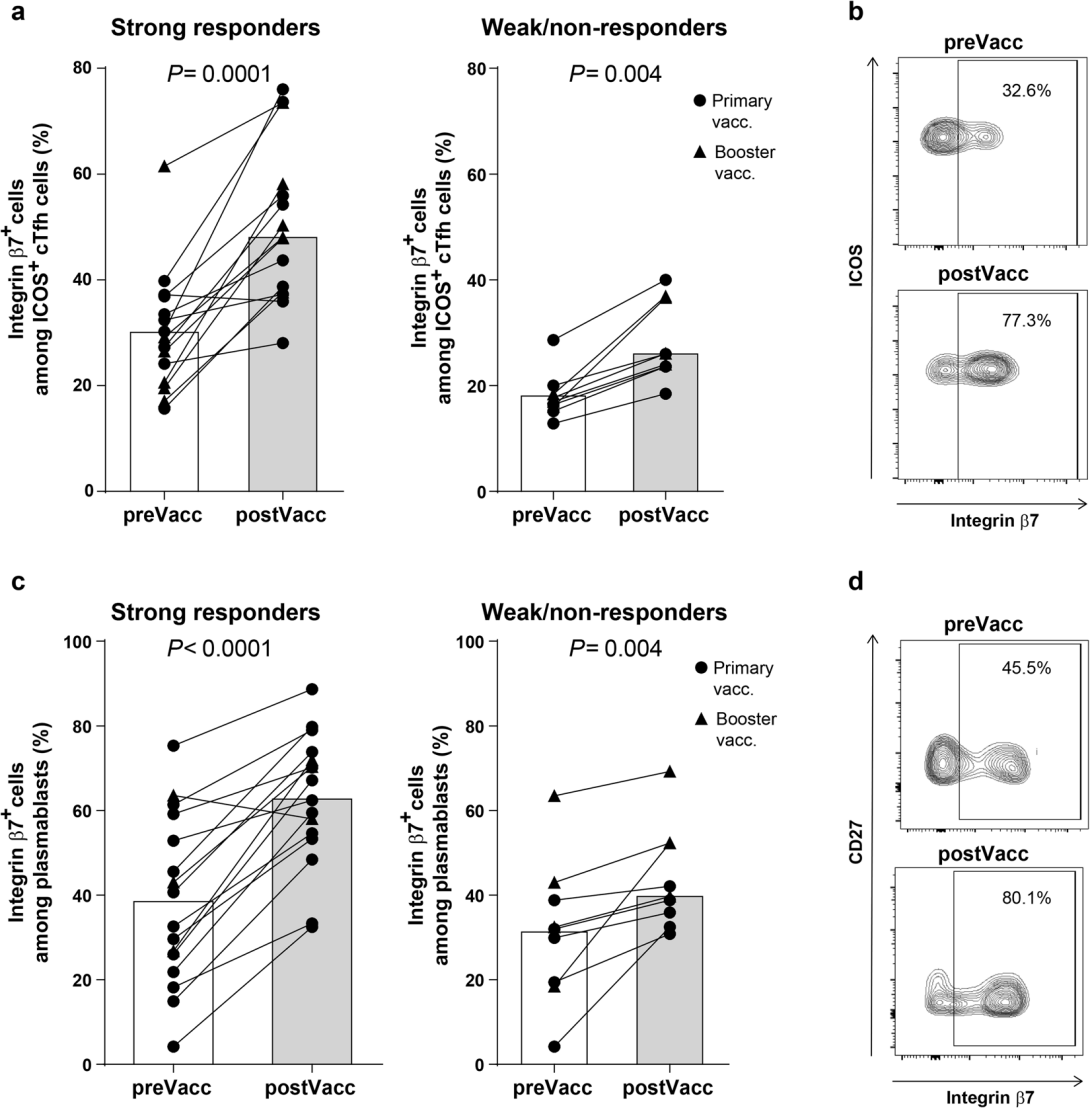
Oral ETEC vaccination induced increased expression of integrin β7 on activated cTfh cells and circulating plasmablasts. Expression of integrin β7 was analysed among activated cTfh cells (ICOS^+^CD4^+^CXCR5^+^) (**a** and **b**) and plasmablasts (CD27^+^CD38^hi^CD19^+^) (**c** and **d**) by FCM in PBMCs collected before (preVacc) and after (postVacc) vaccination. (**a** and **c**) Data pooled from the primary and booster vaccination study. Each symbol represents one individual (the same subjects as in Figs 1a–c and 2a). Bars indicate median values. (**b** and **d**) Representative stainings from one strong ALS responder.

Integrin α4β7 is an important homing receptor recruiting B and T cells to intestinal lymphoid tissue and lamina propria^35^. To evaluate the gut homing properties of cTfh cells activated by ETEC vaccination, the expression of integrin β7 was analyzed on ICOS^+^ cTfh cells from ETEC vaccinees. Among the activated cTfh cells, 15–60% expressed β7 before vaccination and the expression increased significantly in response to both primary and booster vaccinations (Supplementary Fig. S6). The up-regulation of integrin β7 was only observed in activated ICOS^+^ cTfh cells, whereas the frequencies of β7^+^ cells among ICOS^−^ cTfh cells or CD4^+^CXCR5^−^ T cells did not change (Fig. 5a and Supplementary Fig. S6). Interestingly, increased β7 expression was detected on activated cTfh cells from both strong and weak/non-ALS-responders (Fig. 5a).

The expression of β7 also increased on circulating plasmablasts in both strong and weak/non-ALS-responders (Fig. 5c and d). In contrast, vaccination did not influence β7 expression in CD19^+^ B cells or CD19^+^CD27^+^ memory B cells (Supplementary Fig. S6).

In conclusion, these results support increased intestinal homing potential of both cTfh cells and plasmablasts in response to oral ETEC vaccination in a majority of vaccinees.

### Oral vaccination enhanced the expression of Th17 associated markers on activated cTfh cells

**Figure 6:**
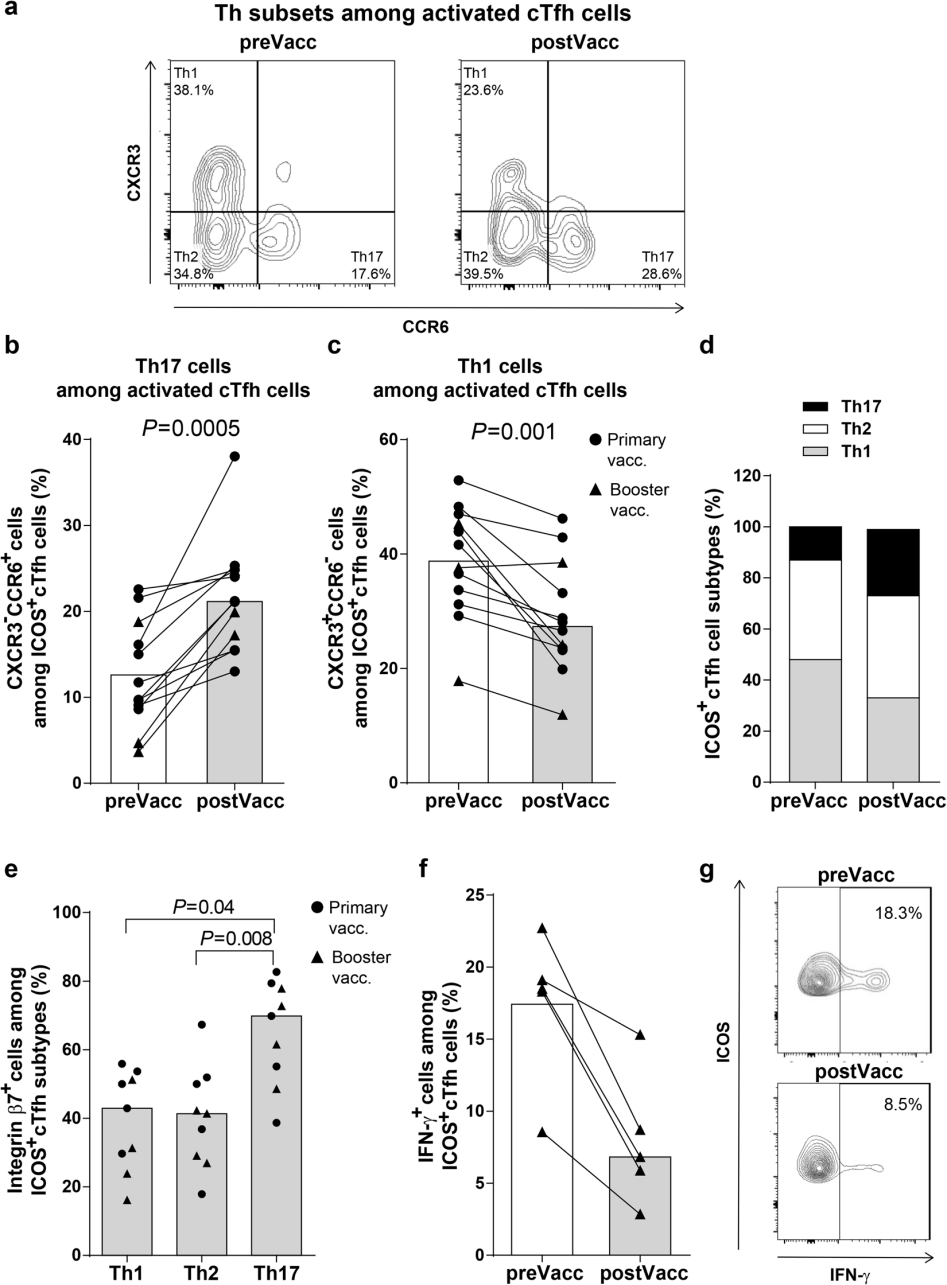
Oral ETEC vaccination induced increased expression of Th17 associated markers on activated cTfh cells. Activated cTfh cells (ICOS^+^CD4^+^CXCR5^+^) were analysed for the expression of CXCR3 and CCR6 by FCM, as illustrated by the representative staining of cells from one strong responder (**a**). Frequencies of activated cTfh cells expressing Th17 (CXCR3^−^CCR6^+^) (**b**) and Th1 (CXCR3^+^CCR6^−^) (**c**) associated markers among strong ALS responders before (preVacc) and after (postVacc) vaccination (n = 12, subset of the 15 strong responders displayed in Figs 1a–c, 2a and 5a and c). (**d**) Proportions of cTfh cells expressing Th1, Th2 and Th17 markers. (**e**) Expression of integrin β7 among activated cTfh cells expressing Th1, Th2 and Th17 markers from strong ALS responders (n = 9). (**f** and **g**) IFN-γ expression among activated cTfh cells from strong responders after stimulation of PBMCs with PMA and ionomycin (n = 5). (**b**,**c**,**e**,**f**) Each symbol represents one individual and bars indicate median values.

Previous studies show that cTfh cells, like non-cTfh CD4^+^ T cells, can be subdivided into Th1, Th2 and Th17 subsets, characterized by production of the signature cytokines IFN-γ, IL-4/IL-5/IL-13 and IL-17A/IL-22, respectively^10,12,32^. These subsets can be distinguished phenotypically by expression of the chemokine receptors CXCR3 and CCR6. Notably, Th17 (CXCR3^−^CCR6^+^) and Th2 (CXCR3^−^CCR6^−^) cTfh cells can efficiently induce both naive and memory B cells to become plasmablasts *in vitro*, while Th1 (CXCR3^+^CCR6^−^) cTfh cells may primarily support memory B cell differentiation^10,12,14,31^. In addition, the Th17 Tfh subset strongly promotes switch to IgA production in naive human B cells^12^. To evaluate whether oral ETEC vaccination influences cTfh cell polarization, we analyzed CXCR3 and CCR6 expression on T cells from strong responders to the ETEC vaccine (Fig. 6a–d). The proportions of activated cTfh cells expressing Th17 markers significantly increased whereas the expression of Th1 markers decreased in response to both primary vaccination and booster vaccination (Fig. 6b–d). The largest increase in expression of Th17 associated markers was observed among activated ICOS^+^ cTfh cells, but a smaller increase was also noted among ICOS^−^ cTfh cells (Supplementary Fig. S7). A very small, but significant increase was also observed among CD4^+^CXCR5^−^ cells. Corresponding decreases in Th1 associated markers were detected in ICOS^+^ and ICOS^−^ cTfh cells as well as the whole CD4^+^ T cell population (Supplementary Fig. S7). Expression of integrin β7 was significantly higher in Th17 compared to Th1 or Th2 subsets (Fig. 6e). Due to the low numbers of weak responders to the vaccine, and the low numbers of ICOS^+^ cTfh cells in this study group, the polarization of ICOS^+^ cTfh cells could not be evaluated among weak vaccine responders.

The down-regulation of Th1 characteristics in cTfh cells in response to vaccination was supported by decreased frequencies of IFN-γ producing cells detected among activated cTfh cells after PMA stimulation in post vaccination samples among all individuals tested (Fig. 6f). In contrast, the production of IFN-γ among ICOS^−^ cTfh cells did not change in response to vaccination. PMA stimulation only induced IL-17A expression in a very small proportion of CD4^+^ T cells which prevented analysis of IL-17A expression in the cTfh subset.

Taken together, these results indicate that ETEC vaccination primarily promotes the development of Th17 associated properties of activated cTfh cells and that these cells have mucosal homing potential.

### cTfh responses to primary vaccination correlated with vaccine specific memory IgA responses

**Figure 7:**
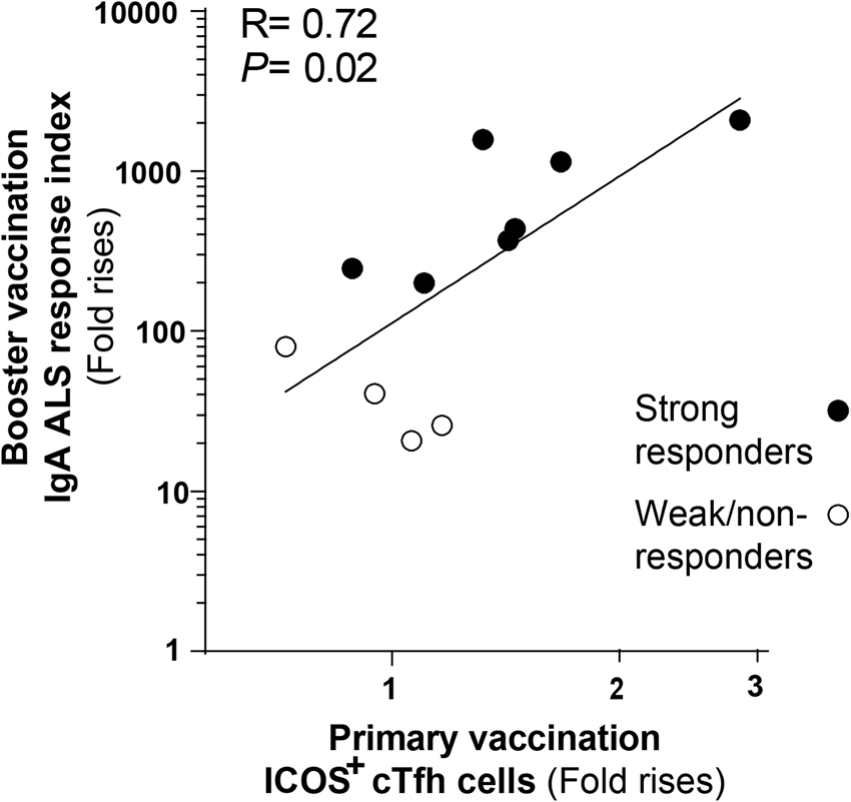
cTfh responses to primary ETEC vaccination correlated with vaccine specific memory IgA ALS responses. Correlation between the cTfh response magnitudes (fold rises in ICOS expression above baseline prevaccination levels) in the primary vaccination study and the combined response index of memory vaccine specific IgA ALS responses against the five major vaccine antigens elicited by booster vaccination. Each symbol represents one individual (n = 11) and data is displayed in a log-log scale plot.

To specifically evaluate the relation between cTfh responses and B cell memory, we investigated the correlation between cTfh responses detected after primary vaccination and the vaccine specific memory IgA responses induced by a late booster dose administered 13–23 months later in all subjects from whom samples were available for analysis both after primary and booster vaccinations. We have recently shown that the strong vaccine specific ALS IgA responses detected on day 4/5 after administration of a single booster ETEC vaccine dose reflect a mucosal memory IgA response, since unvaccinated subjects respond very weakly or not at all to a single dose of vaccine at this early time point^17^. We found that the magnitudes of cTfh responses after primary vaccination correlated significantly with the combined vaccine specific IgA ALS response index after the booster immunization (Fig. 7), i.e. with vaccine specific IgA antibodies produced by plasmablasts derived from mucosal memory B cells reactivated by the booster vaccination and transiently migrating through peripheral blood. These results suggest that activated ICOS^+^ cTfh cells mobilized into peripheral blood early after primary mucosal vaccination may be useful and easily assessable biomarkers for memory B cell induction.

## Discussion

In this study, we have shown that oral vaccination of healthy volunteers resulted in increased frequencies of activated ICOS^+^CD4^+^CXCR5^+^ Tfh-like cells in the peripheral circulation. To our knowledge, this is the first demonstration of a cTfh response in blood after mucosal vaccination, suggesting that monitoring of cTfh cells may provide information about GC responses in the intestinal mucosa. The increased expression of integrin β7 detected on the activated cTfh cells in response to vaccination supports that these cells have gut homing capacity. Importantly, we also showed that the magnitudes of cTfh responses to primary vaccination correlated significantly with both primary and memory intestine-derived vaccine specific IgA responses, providing further support that monitoring of cTfh cells may increase our understanding about how human mucosal B cell responses are generated and maintained after enteric antigen exposure.

Tfh cells can be a limiting factor for the GC reaction and antibody production^36^. B cells compete for Tfh help in the GC, and B cells that are able to efficiently bind and present antigen to Tfh cells are given survival signals, leading to affinity maturation and the formation of long lived plasma blasts and memory B cells^4^. The exact ontogeny of cTfh cells is still unclear, but several lines of evidence suggest that these cells may be committed to the Tfh lineage, but may leave the peripheral lymphoid organ before they enter the GC. Thus, both humans and mice deficient in signaling lymphocytic activation molecule associated protein (SAP), a key molecule for interaction between Tfh cells and B cells in the GC required for Tfh differentiation, have normal proportions of cTfh cells, but low antibody production, supporting that cTfh cells are Tfh precursors^3,13^. This is also suggested by the parallel kinetics of cTfh responses in blood and in lymph node GCs in mice after parenteral vaccination^13^. Thus, cTfh cells may be early lymph node emigrants, spreading to additional lymphoid tissue via the blood.

Our observation of increased integrin β7 expression specifically on activated cTfh cells after oral vaccination suggests that these cells are migrating back to the intestinal mucosa, where the α4β7 receptor binds to mucosal addressin cell adhesion molecule-1 (MAdCAM-1) expressed by endothelial cells in Peyer’s patches (PPs), mesenteric lymph nodes as well as in lamina propria^35^. In mice, a single oral dose of a nitrophenyl-cholera toxin (NP-CT) conjugate was shown to activate B cells in GCs only in a few PPs, but the activated B cell clones then migrated to and expanded in many patches along the intestine in response to repeated oral vaccination^37^. Multiple oral immunizations also resulted in appearance of clonally related plasma cells along the intestinal lamina propria^37^. It is possible that the activated β7^+^ cTfh cells observed in our study may expand and spread between PPs in a similar way, providing more efficient B cell help in multiple PPs upon secondary antigen exposure. We also observed increased β7 expression on plasmablasts in response to ETEC vaccination. Notably, the β7 expression increased on plasmablasts and cTfh cells from almost all vaccinees, including those that responded with low vaccine specific ALS responses, indicating some degree of mucosal activation of these cell subsets also in individuals with low or undetectable ASC responses to the vaccine.

From a practical perspective, our observations that the magnitudes of cTfh responses correlate with vaccine specific IgA responses induced by oral vaccination suggest that cTfh cells may be used to as biomarkers to monitor the development of potentially protective IgA antibody responses in humans. We have recently shown that two doses of the ETEC vaccine administered two weeks apart induced ASC responses to the primary vaccine antigens in a majority of volunteers^16^. In our cTfh analyses, a relatively high proportion of weak or non-responders to the ETEC vaccine were included to allow analysis of correlations between the magnitudes of IgA and cTfh responses. In the whole primary vaccination study, 70% of all subjects would be characterized as strong responders according to our definition. A single oral booster dose of the ETEC vaccine given 1–2 years later induced comparable or slightly increased ASC responses which appeared rapidly upon vaccination, consistent with a mucosal memory response^17^. This booster vaccination study gave us a unique possibility to investigate the correlations between cTfh responses induced by primary vaccinations and vaccine specific memory responses measured years later. Importantly, we detected a significant correlation between the magnitudes of cTfh responses induced within a week after primary vaccinations and the vaccine specific ASC responses elicited by booster vaccination 13–23 months later, which reflect long-lasting mucosal memory. Thus, our data suggest that we may use the cTfh response to primary vaccinations measured shortly after these vaccination as an easily assessable biomarker in order to predict the antigen specific B cell memory remaining several years later. This may have important implications for clinical vaccine studies, which due to practical, cost and time constraints mainly rely on measurements of acute responses, but where memory responses are of critical importance for long lasting protection.

At present, cTfh responses have been monitored after a few different types of parenteral immunizations in humans, including influenza, human papillomavirus, pneumococcal polysaccharide or HIV vaccinations^14,15,29,30,34^. Similar to our findings, increased frequencies of ICOS expressing cTfh cells were observed in the circulation after such parenteral vaccinations^14,15,29,30,34^. Expanded populations of ICOS^+^CD4^+^CXCR5^+^ T cells have also been observed both in blood and draining lymph nodes in mice after parenteral vaccination with NP-ovalbumin^38^. cTfh responses to vaccination have also been analyzed using alternative combinations of cell surface markers. A majority of activated ICOS^+^CD4^+^CXCR5^+^ cells in blood are CCR7^low^ PD-1^high^, whereas resting cTfh cells, representing the majority of cTfh cells, are ICOS^−^ CCR7^low/int^ PD-1^−/int ^^10^. Using the CCR7^low^ PD-1^high^ phenotype to distinguish cTfh with efficient effector functions, He *et al*. demonstrated cTfh responses to influenza vaccination with similar kinetics as reported using ICOS to define activated cTfh cells^13^. A corresponding expanded subset of CCR7^low^ PD-1^high^ cells capable of maturing into mature and functional GC Tfh cells upon antigen reexposure was also observed in peripheral blood of mice after parenteral vaccination^13^. In our hands, analysis of ICOS expression gave more consistent results than analysis of the CCR7^low^ PD-1^high^ cell population, but similar trends were obtained in subjects where cell numbers allowed parallel analysis of all markers. Recently, circulating Tfh-like cells in humans and non-human primates have also been analyzed using expression of other activation markers including OX40, CD25, CD154 and/or IL-21 after *in vitro* stimulation with specific antigens^39–41^. However, such protocols require large numbers of cells, which were not available in the present study.

Notably, we observed an expansion of Th17-like cells (CXCR3^−^CCR6^+^), identified to be the subset with most potent B cell helping capacity^10,12,14,31,32^, among the activated cTfh cells in strong ALS responders after vaccinations, with a parallel decrease in the Th1 (CXCR3^+^CCR6^−^) subset as well as reduced IFN-γ production. This is in contrast to influenza and human papillomavirus vaccination, where Th1 cTfh cells expanded^14,29^, and may result from the differences in antigen delivery route or the viral *versus* bacterial source of antigens. The Th1 cTfh subset has also been reported to be preferentially expanded in HIV and malaria infections^42,43^. Since Th1 cTfh cells, in contrast to Th17 or Th2 cTfh cells, are not able to induce naïve B cells to produce antibodies and to switch isotypes^10,12,14,31^, it has been suggested that poor/unprotective antibody responses to influenza and malaria may be due to preferential activation of Th1 Tfh cells^14,42^. The Th17 type subset of cTfh cells is also reduced in patients with some genetic immune system disorders, including *STAT3* and *STAT1* mutations^32^, which has been suggested to contribute to the low antibody levels and low frequencies of memory B cells in observed in such patients^44,45^. Thus, it is encouraging that the ETEC vaccine induced expansion of the Th17 cTfh subset, which can strongly promote IgA and IgG production in both naïve and memory B cells and which seems to be the most efficient inducer of IgA responses among the different cTfh subsets^10,12,32^. It is also noteworthy that the Th17 cTfh cells had the highest integrin β7 expression of all cTfh subsets. Similar expansion of the Th17 and decrease of the Th1 subset was observed after primary and booster vaccination, indicating that Th17 cells are preferentially promoted both in naïve and previously primed subjects. Interestingly, the live rVSV-ZEBOV parenteral Ebola vaccine candidate, which showed protection in a phase III efficacy trial in the Guinea, was recently also demonstrated to induce Th17 polarization among cTfh cells^41^. Furthermore, Th17 polarization of cTfh cells has also been associated with production of broadly neutralizing antibodies in a subgroup of HIV patients^31^, providing further support for an important role of Th17-like cTfh cells in promoting protective humoral immune responses.

Previous studies show that the Th17 cTfh cells express the transcription factor RORγT and produce the Th17 cytokines IL-17A and IL-22^12,32^. This cell subset is also a potent producer of IL-21, which is essential for the ability to promote affinity maturation, plasmablast formation and development of memory B cells^11,12,32^. We observed increased production of IL-21 and decreased production of IFN-γ in response to ETEC vaccination in ICOS^+^ cTfh cells and demonstrated that vaccination also enhanced the capacity of CXCR5^+^CD4^+^ T cells to promote B cell survival, plasmablast formation and IgA and IgG production. After vaccination, CXCR5^+^CD4^+^ T cells were also able to support LTB-specific antibody production from cocultured memory B cells in two subjects where cell numbers and sample volumes allowed such analyses. The limited cell numbers available prevented a more detailed characterization of the phenotype and function of cTfh cells before and after vaccination. It therefore remains to be determined if the increased helper function observed after vaccination is due to increased proportions of activated ICOS^+^ cTfh cells in the cultures, altered cytokine production in the cTfh cells and/or altered composition of the cocultured memory B cells. It is also important to note that the limited ICOS expression and helper capacity observed among CXCR5^−^ cells may have been a result of high proportions of naïve CD4^+^ T cells in this cell population. In future studies, it will thus be important to compare the function of ICOS^+^ and ICOS^−^ memory cTfh cells more specifically after oral vaccination and to perform more extensive coculture experiments with cTfh cell subsets in combination with both naïve B cells and memory B cells collected before compared to after vaccination. Since the ETEC vaccine was highly immunogenic, the number of vaccinees with weak ALS responses was also relatively small, limiting the possibilities to perform extensive analysis cTfh in this subject group in our study. It will thus also be of interest to compare the function and polarization of the cTfh T cell subsets in strong and weak ALS responders in continued studies.

Previous studies in mice demonstrate that dmLT and the related native enterotoxin adjuvants cholera toxin (CT) or ETEC LT can strongly promote production of mucosal antibodies^2,18^ and CT have also been shown to drive GC reactions and memory B cell development^46^. However, there were no apparent differences in the magnitudes of cTfh responses or expression of Th1, Th2 or Th17 associated markers in subjects immunized with or without dmLT adjuvant in our study, although this needs to be confirmed in a larger number of volunteers in later trials. Our recent results in adults suggest that dmLT only enhanced magnitudes of ASC responses to the antigens present in the lowest amounts in the ETEC vaccine, particularly CS6^16^, which is consistent with the dose sparing effect of dmLT observed in mice^18^, and that dmLT did not affect memory development^17^. Since a high dose of the vaccine was administered in this study, it is possible that the adjuvant was not able to further promote Tfh and B cell responses to most antigens. In continued trials testing the immunogenicity and efficacy of the ETEC vaccine with and without dmLT adjuvant, it will be important to specifically monitor the Th17 cTfh subset, since *in vitro* as well as *in vivo* data support that dmLT may promote Th17 responses^47–49^, and, as previously mentioned, the Th17-like cTfh subset has been shown to be the most potent inducer of IgA responses *in vitro*^12^ and Th17 polarization of cTfh cells has been associated with induction of protective antibody responses in different settings^31,41^.

In conclusion, we have shown that oral vaccination with a killed whole cell ETEC vaccine induced the appearance of ICOS^+^ activated Tfh-like T cells with a Th17 and gut homing phenotype in peripheral blood. cTfh cells from vaccinees had potent B cell helping capacity and could promote both total and vaccine specific IgA production. Our demonstration of functional and gut homing cTfh response to ETEC vaccination indicates that the vaccine induces potent GC reactions in the intestinal mucosa. Our observation that the magnitudes of primary cTfh cell responses correlate with both primary and memory vaccine specific mucosal IgA responses provide further support of a functional role of these cells and also suggest that cTfh responses may be used as early and easily assessable biomarkers to predict long-lived memory responses induced by mucosal vaccination in humans.

## Methods

### Immunizations and sample collection

Detailed descriptions of the two clinical vaccine trials have been published elsewhere, together with results from the primary endpoint analyses of these trials^16,17^. Briefly, in the primary vaccination study previously unvaccinated Swedish adults received two oral doses of the ETVAX ETEC vaccine with or without 10 or 25 µg dmLT and in the booster vaccination study, random subsets of subjects previously immunized with ETVAX with or without 10 µg dmLT received a single oral booster dose of vaccine alone 13–23 months later (See supplementary Fig. S1 for additional information about the study design and sample collection schedules). PBMCs were isolated from venous blood by density-gradient centrifugation using Ficoll-Paque and cryopreserved as described by Kreher *et al*.^50^.

All studies were approved by the Ethical Review Board in the Gothenburg Region and the Swedish Medical Product Agency. Written informed consents were obtained from all volunteers before participation. All experiments and procedures were performed according to relevant guidelines and regulations. The clinical trials are registered in the ISRCTN data base (ISRCTN91363076 and ISRCTN27096290). Preliminary data from the present study was presented at the Vaccines for enteric diseases (VED) conferences in Edinburgh 2015 and in Albufeira 2017 and the Correlates of enteric vaccine-induced protection meeting (Fondation Merieux) in Annecy 2016^51^.

### Vaccine

The multivalent ETEC vaccine ETVAX consists of four inactivated recombinant *E*. *coli* strains which overexpress the CFs CFA/I, CS3, CS5 and CS6, respectively, mixed with LCTB*A*, a recombinantly produced LTB/cholera toxin B subunit hybrid protein^16,18^. The vaccine was administered orally alone or together with 10 or 25 µg dmLT adjuvant in 150 ml bicarbonate buffer. dmLT (R192G/L211A) is an LT-derived protein which contains two genetic substitutions in the A subunit which eliminates the enterotoxic activity without removing the adjuvant activity^16,52^.

### ALS analyses

Previously published results from ALS analyses were used for comparisons between ASC and cTfh responses in this paper^16,17^. Briefly, 2 × 10^6^ fresh PBMCs per well were cultured in 96-well plates in the ALS assay and supernatants were collected after 72 hours^16,17,25^. Antibody levels in ALS specimens were analyzed by ELISA using plates coated with CFA/I, CS3, CS5 or CS6 or with GM1 plus LTB^16,17^.

### Study subjects

PBMCs isolated from 14 subjects, aged 18–32 years (median 24 years, 5 females) in the primary vaccination study and 10 subjects in the booster vaccination study (separate from the subjects evaluated after primary vaccination) aged 22–45 years (median 26 years, 8 females) were included in the basic analyses presented in Figs 1, 2, 5 and 6 and the corresponding supplementary figures in the present study (total n = 24). Subjects were selected based on the availability of frozen cells and their combined ALS response index calculated from ALS measurements reported previously^16,17^. Subjects with a combined ALS response index, defined as the sum of the magnitudes (maximal fold rises above the prevaccination baseline) of IgA ALS responses to the five major vaccine antigens LTB, CFA/I, CS3, CS5 and CS6, of ≥150 were defined as strong responders and subjects with an index ≤ 100 as weak/non-responders (Supplementary Fig. S1). Additional subjects from the primary vaccination study (n = 7, median age 24, 20–31 years, 3 females) and the booster vaccination study (n = 7, median age 25, 23–30 years, 3 females) were included in functional (Figs 1f, 4 and 6f) and correlation (Figs 3 and 7) analyses.

### Flow cytometric analysis

FCM analysis was performed using an LSRII flow cytometer (BD Pharmingen, San Diego, CA) and FlowJo analysis software (Version 10, Treestar Inc, Ashland, OR).

For phenotypic FCM analyses of cTfh cells, cryopreserved PBMCs (2 × 10^6^ cells/sample) were stained with anti-CD3-Pacific Blue (clone UCHT1), anti-CD4-APC Cy7 (RPA-T4), anti-CXCR5-Alexa Fluor 488 (RF8B2), anti-ICOS-PerCPCy5.5 (DX29), anti-CCR6-PECy7 (11A9), anti-CXCR3-Alexa Fluor 700 (1C6) anti-Integrin β7-APC (FIB504), anti-CD27-Alexa Fluor 700, anti-CD45RO-PECy7 (UCHL1), anti-CCR7- PECy7 (150503), anti-CD62L-FITC (SK11) (all from BD Biosciences, San Jose, CA) and anti-PD-1-PE (EH12.2H7, BioLegend, San Diego, CA). The anti-integrin β7 antibody clone used (FIB504) has been shown to recognize the same population of human B cells as detected by the α4β7 monoclonal antibody clone ACT-1^24^.

For B cell characterization, PBMCs were stained with anti-CD3-Pacific Blue (for exclusion of T cells), anti-CD19-APCCy7 (H1B19, BioLegend, San Diego, CA), anti-CD38-PerCPCy5.5 (HIT2), anti-CD27-Alexa Fluor 700 (M-T271), anti-Integrin β7-APC (all from BD Biosciences, San Jose, CA) and anti-IgA-PE (IS11–24D, Miltenyi Biotec, Bergisch Gladbach, Germany). Staining with anti-CD20-PE (2H7; BD Biosciences, San Jose, CA) was included in control samples (Supplementary Fig. S3).

Aqua fluorescent live/dead (Fixable Dead Cells Stain Kit, Invitrogen Life Technologies, Carlsbad, CA) was included in all panels for exclusion of dead cells. More than 95% of the cells were alive in all phenotypic experiments, as determined by live/dead staining and trypane blue exclusion. At least 500 000 events in the lymphocyte gate were acquired per sample as defined by forward and side scatter. Control experiment verified that comparable results were obtained using fresh and cryopreserved cells and for intra- and extracellular staining of IgA.

For evaluation of cytokine production by FCM, cryopreserved PBMCs were thawed and cultured in DMEM (Gibco-Invitrogen Life Technologies, Carlsbad, CA) with 5% AB+ serum and 0.1 mg/ml gentamicin at 37 °C. Cells were stimulated with 50 ng/mL of PMA and 1 μg/mL of ionomycin in the presence of brefeldin A (10 μg/mL) for 5 hours. After live/dead staining, PBMCs were stained with anti-CD3-Alexa Fluor 700 (UCHT1), anti-CD4-APCCy7 (RPA-T4), anti-CXCR5-Alexa Fluor 647 (RF882) and anti-ICOS-PerCPCy5.5, the cells were fixed, permeabilized (Fixation/Permeabilization Solution Kit, BD Biosciences, San Jose, CA), and stained intracellularly with anti-IL-21-PE (eBio3A3-N2), anti-IFN-γ-V450 (B27) and anti-IL-17-PECy7 (eBio64DEC17) (all from eBiosciences, San Diego, CA).

### Cell sorting and coculture experiments

To sort cTfh and memory B cells for coculture experiments, cryopreserved PBMCs were stained with anti-CD3-Pacific Blue, anti-CD4-Alexa 700 (RPA-T4), anti-CXCR5-Alexa Fluor 647, anti-CD27-PE (M-T271) (all from BD Biosciences, San Jose, CA) and anti-CD19-APCCy7. Aqua fluorescent live/dead was included for exclusion of dead cells. cTfh (CD3^+^CD4^+^CXCR5^+^) cells and memory B cells (CD3^−^CD19^+^CD27^+^) were sorted (Supplementary Fig. S5) using a BD FACSAria IIIu cell sorter (BD Pharmingen). The purity of the sorted cell populations was always ≥94%.

Sorted CD4^+^CXCR5^+^ or CD4^+^CXCR5^−^ cells (2 × 10^4^ cells/well) were cocultured with CD19^+^CD27^+^ memory B cells (2 × 10^4^ cells/well) in RPMI 1640 medium supplemented with 50 µg/mL penicillin/streptomycin/gentamycin, 1 mM sodium pyruvate, 0.1 nM non-essential amino acids, 50 μM β-mercaptoethanol and 10% heat inactivated fetal calf serum (all from Gibco-Invitrogen Life Technologies, Carlsbad, CA) in the presence of 1 μg/ml SEB in 96-well U-bottomed plates, as described^14^. Culture supernatants were collected after 6 days and the presence of CD3^−^CD19^+^CD27^+^CD38^hi^ IgA^+^ plasmablasts were analyzed among the recovered cells after staining with with anti-CD3-Pacific Blue, anti-CD4-FITC (RPA-T4), anti-CXCR5-Alexa Fluor 647, anti-CD27-Alexa Fluor 700, anti-CD38-PerCPCy5.5 (all from BD Biosciences, San Jose, CA), anti-CD19-APCCy7 and anti-IgA-PE. Total IgA and IgG concentrations in culture supernatants were measured by human Ready-SET-Go! ELISA (eBioscience, San Diego, CA). LTB-specific IgA titers were measured using ELISA plates coated with GM1 ganglioside plus LTB, as decribed^16,17,25^.

### Statistical analyses

Data was evaluated with GraphPad Prism (Version 6.0, GraphPad Software, Inc.). Wilcoxon signed rank test was used to evaluate differences between pre- and postvaccination samples and for evaluation of β7 expression in different cTfh subsets (with Bonferroni correction, when applicable). Spearman test was used for correlation analyses. *P* values of <0.05 were regarded as significant.

### Data availability

All data generated or analysed during this study are included in this article and its supplementary information files.

## Electronic supplementary material

Supplementary figures
