# Supplementary material for: Activated T follicular helper-like cells are released into blood after oral vaccination and correlate with vaccine specific mucosal B-cell memory
Source: Sci Rep. 2018 Feb 9;8:2729. doi: 10.1038/s41598-018-20740-3 (PMC5807513; doi:10.1038/s41598-018-20740-3)
Supplement: Supplementary file 1 — Supplementary figures [file 41598_2018_20740_MOESM1_ESM.pdf]

Supplementary figures

Activated T follicular helper-like cells are released into blood after oral vaccination and correlate with vaccine specific mucosal B-cell memory

Ana Cárdeno, Maria K. Magnusson, Marianne Quiding-Jarbrink and Anna Lundgren

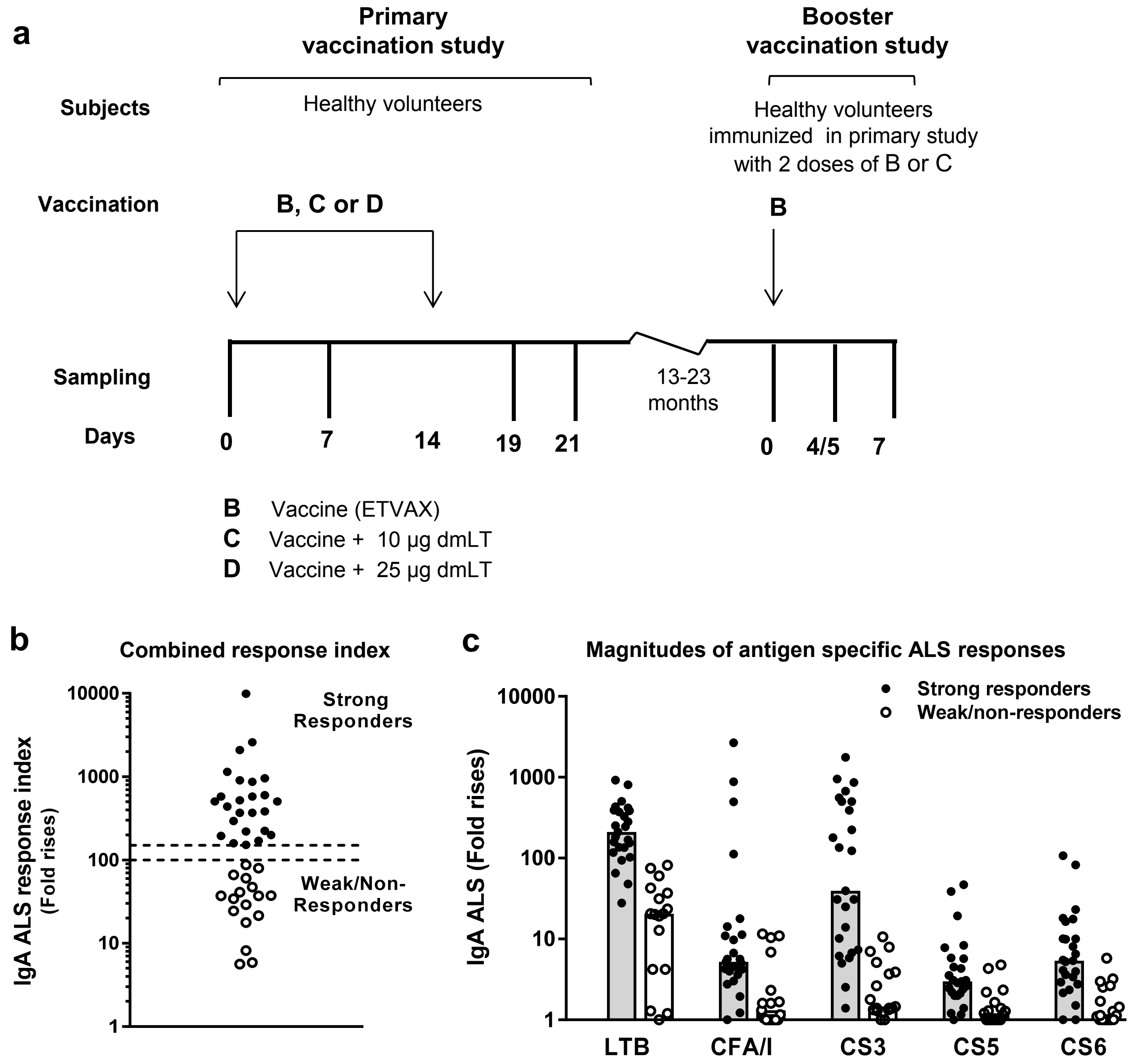

Supplementary figure S1. Study design. (**a**) In the primary vaccination study, subjects were immunized with two oral doses of the inactivated ETEC vaccine (ETVAX) only (Group B), vaccine + 10 µg dmLT (Group C) or vaccine + 25 µg dmLT (Group D) 14 days apart (Group A received placebo and was not included in this study). PBMCs were isolated before (day 0) and after (day 7, 19 and 21) administration of the first vaccine dose. In the booster vaccination study, a subset of subjects from Group B and C in the primary vaccination study received a single late booster vaccine dose alone 13-23 months after the primary vaccinations. PBMCs were isolated before (day 0) and after (day 4/5 and 7) administration of the booster vaccine dose. (**b** and **c**) Samples used for this study were selected based on the magnitudes of IgA ALS responses (fold rises; maximal IgA antibody levels measured at any time point after one or two vaccinations divided by prevaccination levels) against the five major vaccine antigens (LTB, CFA/I, CS3, CS5 and CS6) and the availability of frozen cells. The magnitudes of IgA ALS responses against each individual antigen after primary or booster vaccination, respectively, were summarized in a combined magnitude response index for each subject. (**b**) Strong ALS responders were defined as subjects with a combined magnitude index ≥150 and weak/non responders had an index ≤100, as illustrated here for the 14 subjects from the primary vaccination study and the 10 subjects from the booster vaccination study included in the basic analyses (total n=24) displayed in Fig. 1, 2, 5 and 6 (subset) and corresponding supplementary Fig. **S4**, **S6** and **S7** (subset). (**c**) Magnitudes of ALS IgA responses against the five different major vaccine antigens in the same 24 subjects.

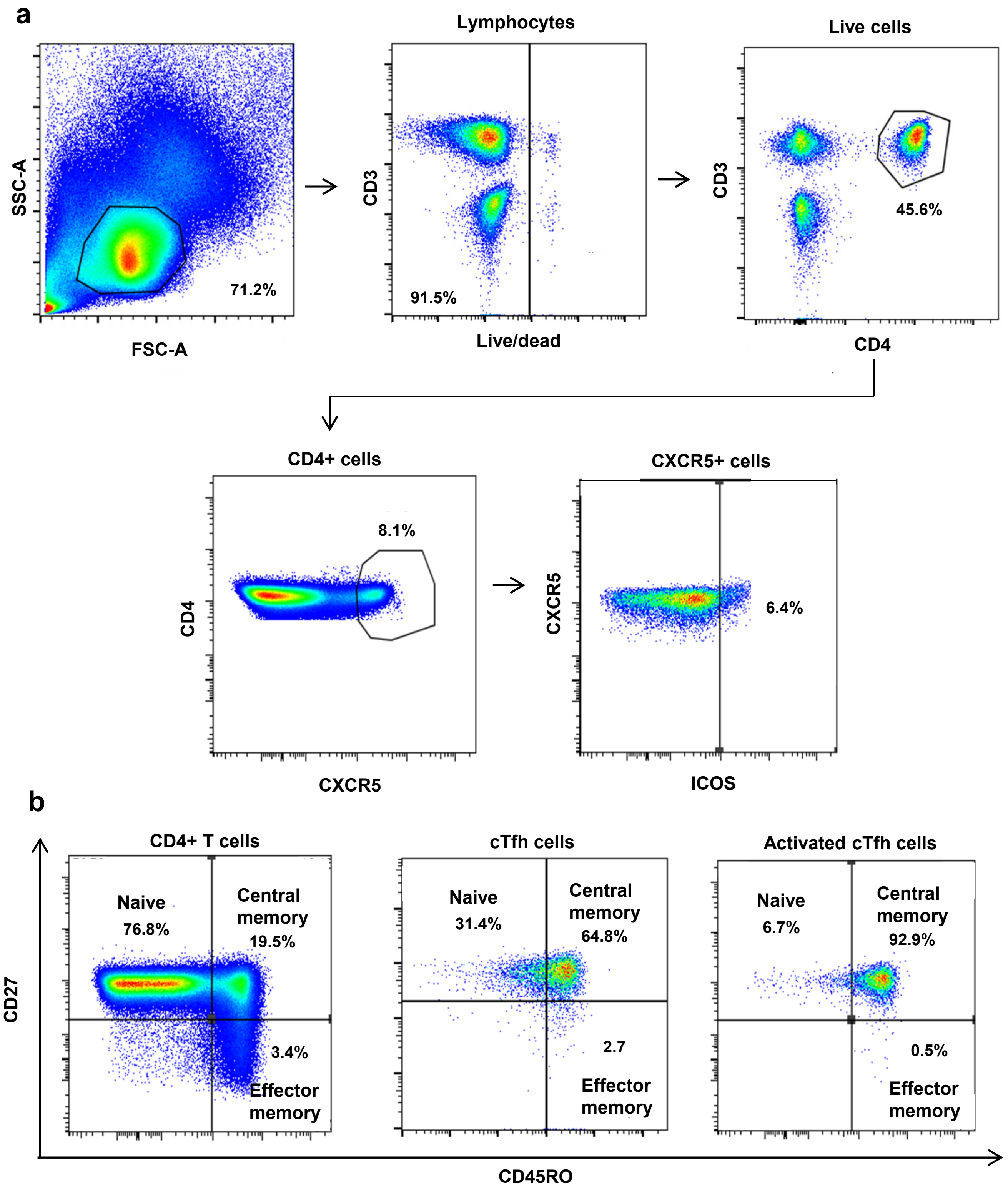

Supplementary figure S2. Gating strategy for phenotypic analysis of cTfh cells. Thawed cryopreserved PBMCs were stained for FCM analysis. (**a**) cTfh cells were defined as $CXCR5^{+}$ cells among $CD3^{+}CD4^{+}$ live lymphocytes and activated cTfh cells were defined as $ICOS^{+}$ cells among $CD3^{+}CD4^{+}CXCR5^{+}$ live lymphocytes. (**b**) The expression of CD27 and CD45RO, differentiating central memory ($CD45RO^{+}CD27^{+}$), effector memory ($CD27^{-}CD45RO^{+}$) and naive ($CD27^{-}CD45RO^{-}$) cells, was analysed on $CD3^{+}CD4^{+}$ T cells, cTfh ($CD3^{+}CD4^{+}CXCR5^{+}$) and activated cTfh ($CD3^{+}CD4^{+}CXCR5^{+}ICOS^{+}$) cells.

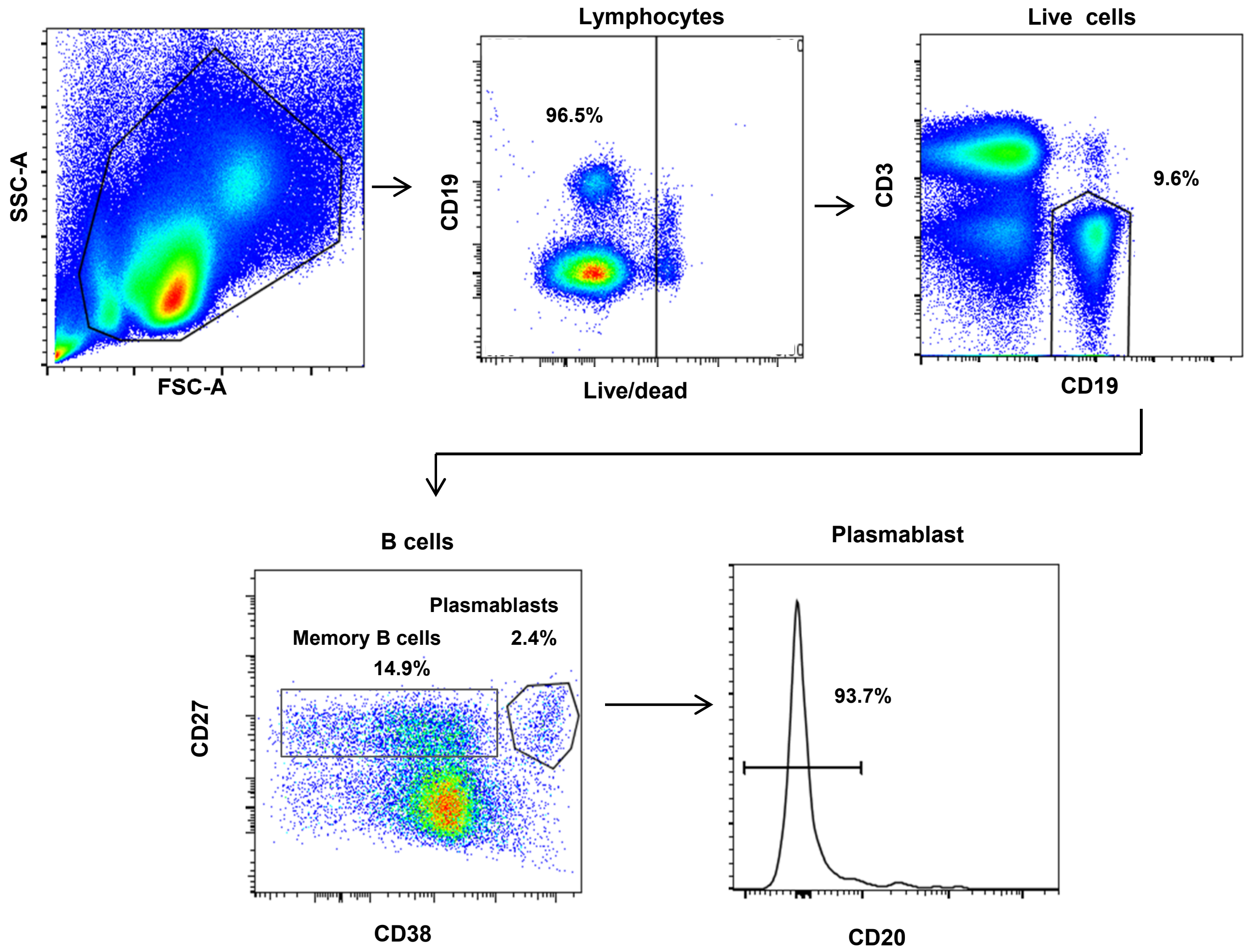

Supplementary figure S3. Gating strategy for phenotypic analysis of B cells and plasmablasts. Thawed cryopreserved PBMCs were stained for FCM analysis. B cells were defined as $CD3^{-}CD19^{+}$ cells among live cells and memory B cells and plasmablasts as $CD27^{+}$ or $CD27^{+}CD38^{hi}$ cells, respectively, among B cells. CD20 expression was analyzed in $CD3^{-}CD19^{+}$ $CD27^{+}CD38^{hi}$ cells to verify the plasmablast phenotype.

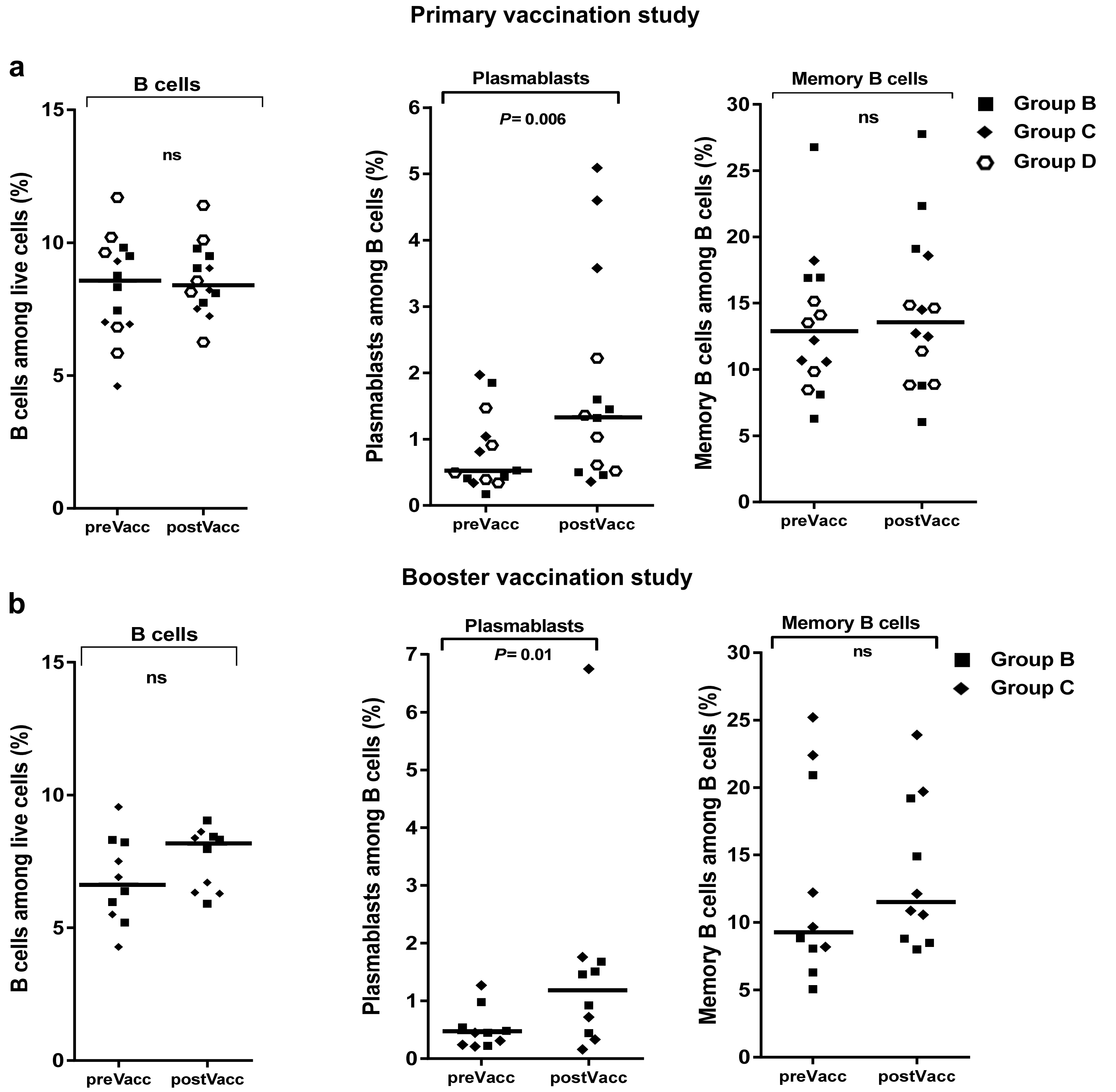

Supplementary figure S4. Frequencies of circulating B cells and plasmablasts before and after ETEC vaccination. PBMCs were analysed for the presence of total B cells ($CD3^-CD19^+$ cells among live cells); memory B cells ($CD27^+$ cells among B cells) and plasmablasts ($CD27^+CD38^{hi}$ cells among B cells) by FCM before (preVacc) and after (postVacc) primary (**a**, n=14) and booster vaccination (**b**, n=10). Each symbol represents one individual (same subjects as displayed in Fig. **2a**) and lines indicate median values.

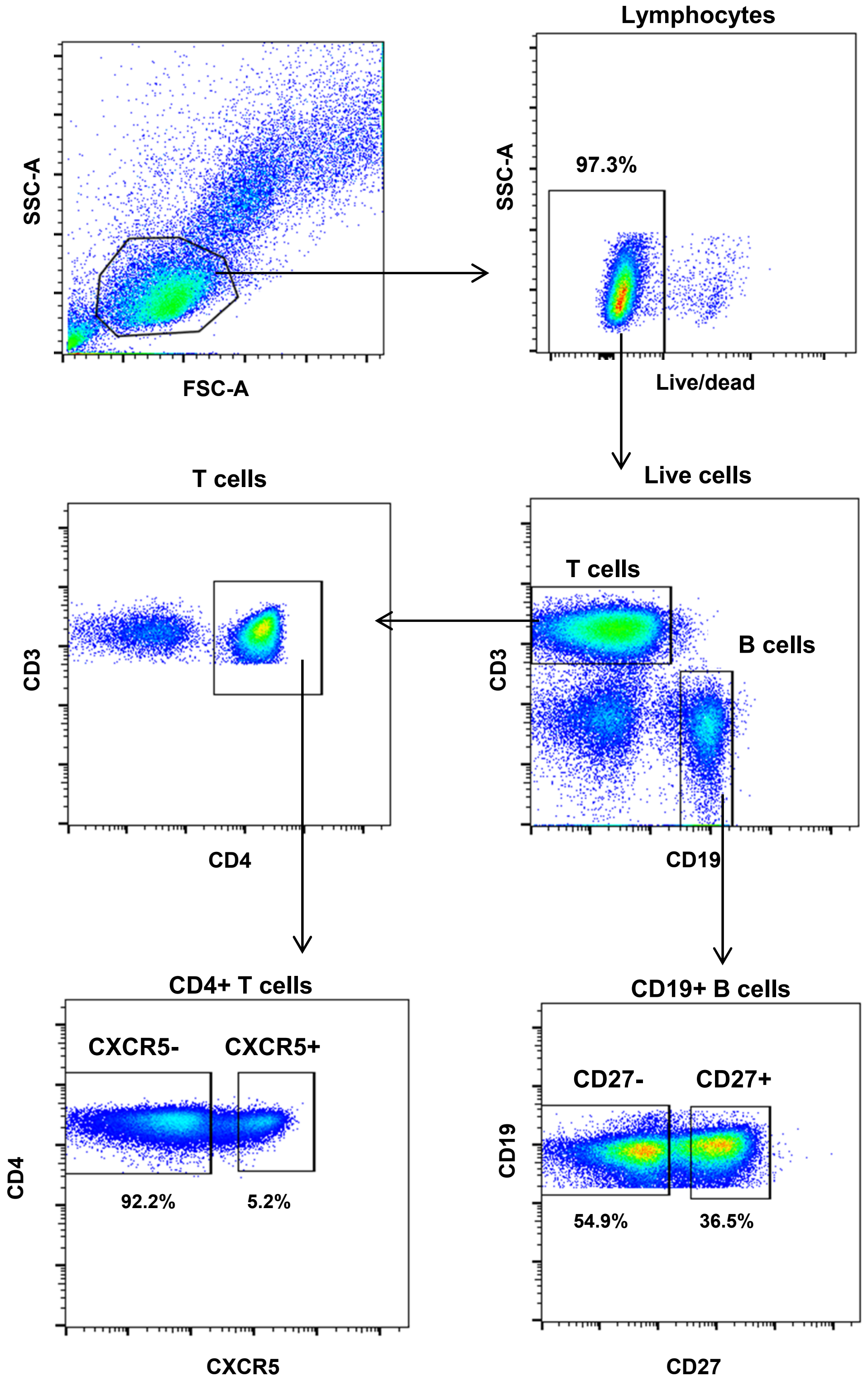

Supplementary figure S5. Strategy for sorting of cTfh, non-cTfh and memory B cells for coculture experiments. $CD3^+CD4^+CXCR5^+$ cTfh cells, $CD3^+CD4^+CXCR5^-$ and $CD19^+CD27^+CD3^-$ memory B cells were sorted from live PBMCs as indicated in the representative FCM plots.

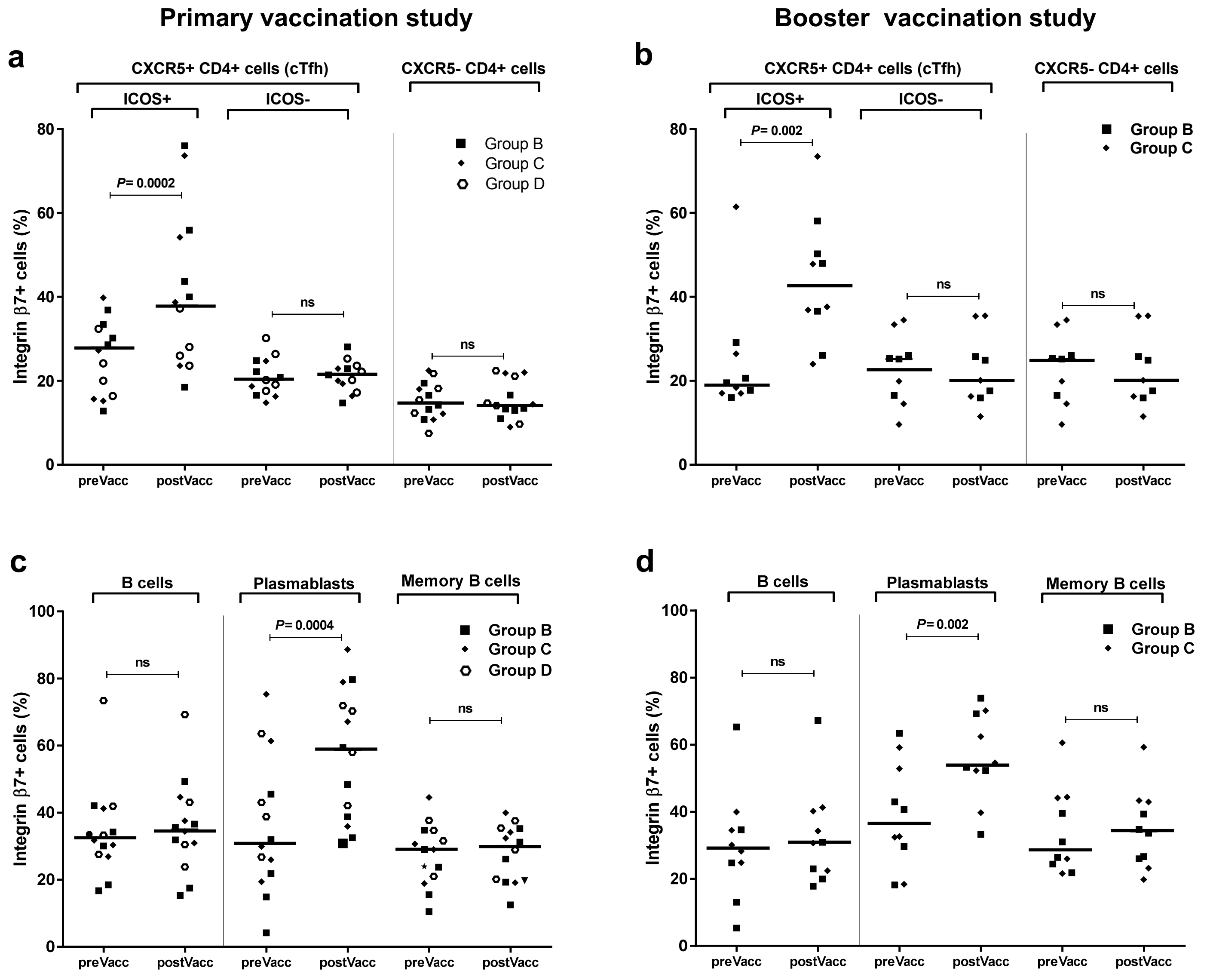

Supplementary figure S6. Expression of integrin β7 on activated cTfh cells and plasmablasts before and after ETEC vaccination. (**a** and **b**) Integrin β7 expression was analyzed among activated cTfh ($ICOS^+CD4^+CXCR5^+$), nonactivated cTfh ($ICOS^-CD4^+CXCR5^+$) and $CD4^+CXCR5^-$ T cells before (preVacc) and after (postVacc) primary (**a**) and booster vaccination (**b**). (**c** and **d**) Integrin β7 expression was also determined among B cells ($CD3^-CD19^+$), plasmablasts ($CD3^-CD27^+CD38^{hi}$) and memory B cells ($CD3^-CD19^+CD27^+$) before and after primary (**c**) and booster vaccination (**d**). (**a-d**) Each symbol represents one individual (same subjects as displayed in Fig. **5a** and **5c**) and lines indicate median values.

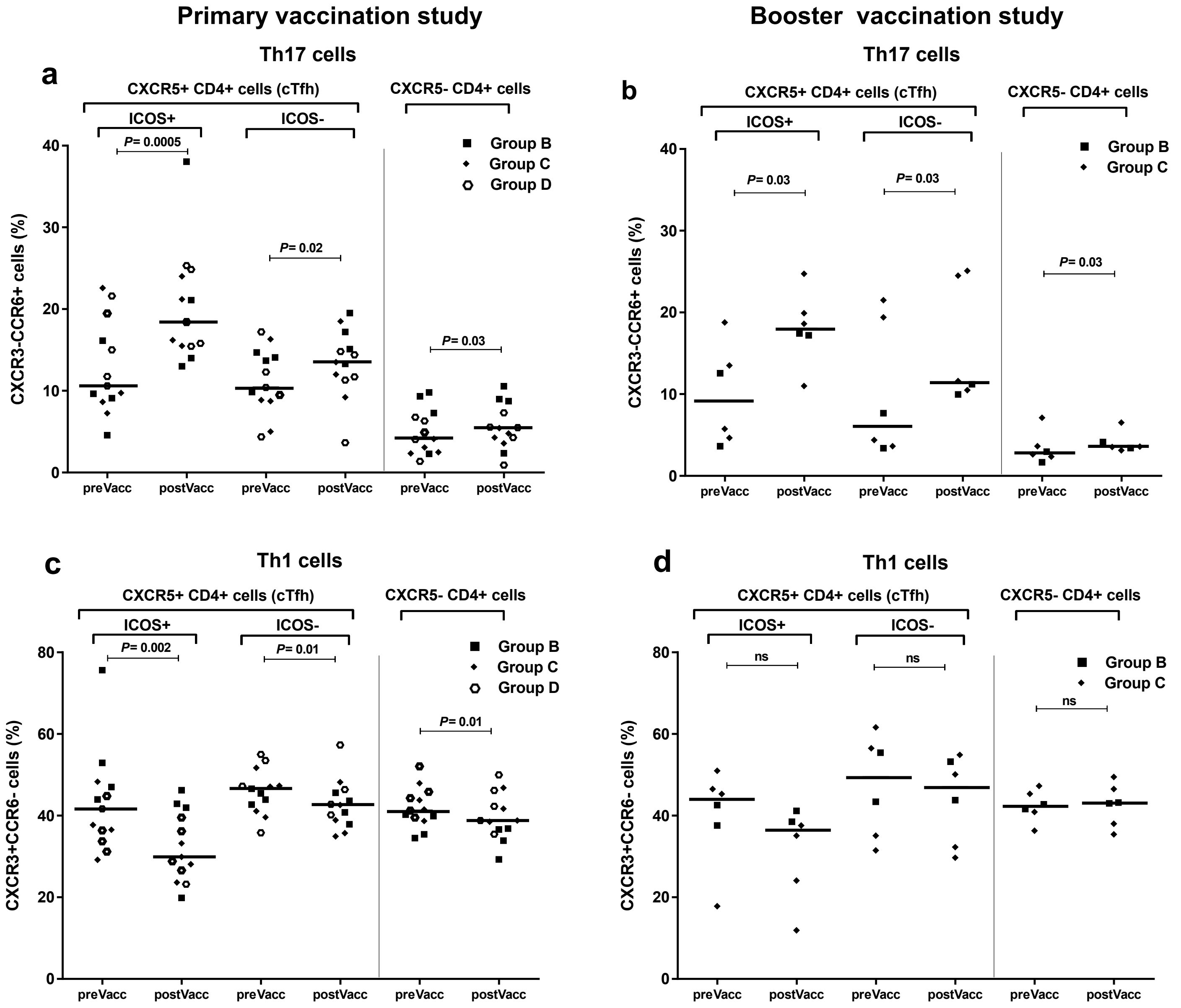

Supplementary figure S7. Expression of Th1 and Th17 associated markers on cTfh and non-cTfh cells after ETEC vaccination. Th17 ($CXCR3^-CCR6^+$; **a** and **b**) and Th1 ($CXCR3^+CCR6^-$; **c** and **d**) associated markers were analysed among activated cTfh ($ICOS^+CD4^+CXCR5^+$), non-activated cTfh ($ICOS^-CD4^+CXCR5^+$) and $CD4^+CXCR5^-$ T cells before (preVacc) and after (postVacc) primary (**a** and **c**, n=13) and booster vaccination (**b** and **d**, n=6). Each symbol represents one individual and lines indicate median values.
